# Delayed Chest Closure for Oversized Lung Allograft in Lung Transplantation: a Retrospective Analysis from Turkey

**DOI:** 10.21470/1678-9741-2020-0299

**Published:** 2021

**Authors:** Ali Yeginsu, Ahmet Erdal Tasci, Mustafa Vayvada, Bulent Aydemir, Nigar Halis, Atakan Erkilinç, Sevinc Citak, Ersin Cardak

**Affiliations:** 1 Department of Thoracic Surgery, Yeni Yuzyil University School of Medicine Gaziosmanpasa Hospital, Istanbul, Turkey.; 2 Department of Thoracic Surgery, University of Health Sciences Kartal Kosuyolu Yuksek Ihtisas Hospital, Istanbul, Turkey.; 3 Department of Thoracic Surgery, Siyami Ersek Thoracic and Cardiovascular Training and Research Hospital, Istanbul, Turkey.; 4 Department of Anesthesia and Reanimation, Kartal Kosuyolu Yuksek Ihtisas Hospital, Istanbul, Turkey.; 5 Department of Thoracic Surgery, Sureyyapasa Chest Disease and Thoracic Surgery Training and Research Hospital, Istanbul, Turkey.

**Keywords:** Surgical Wound Infection, Reoperation, Airway Extubation, Lung Transplantation, Total Lung Capacity, Tissue Donors, Thorax, Intensive Care Units, Allografts.

## Abstract

**Introduction:**

The aim of this study was to evaluate the delayed chest closure (DCC) results in patients who underwent lung transplantation.

**Methods:**

Sixty patients were evaluated retrospectively. Only bilateral lung transplantations and DCC for oversized lung allograft (OLA) were included in the study. Six patients who underwent single lung transplantation, four patients who underwent lobar transplantation, two patients who underwent retransplantation, and four patients who underwent DCC due to bleeding risk were excluded from the study. Forty-four patients were divided into groups as primary chest closure (PCC) (n=28) and DCC (n=16). Demographics, donor characteristics, and operative features and outcomes of the patients were compared.

**Results:**

The mean age was 44.5 years. There was no significant difference between the demographics of the groups (*P*>0.05). The donor/recipient predicted total lung capacity ratio was significantly higher in the DCC group than in the PCC group (1.06 *vs*. 0.96, *P*=0.008). Extubation time (4.3 *vs*. 3.1 days, *P*=0.002) and intensive care unit length of stay (7.6 vs. 5.2 days, *P*=0.016) were significantly higher in the DCC group than in the PCC group. In the DCC group, postoperative wound infection was significantly higher than in the PCC group (18.6% vs. 0%, *P*=0.19). Median survival was 14 months in all patients and there was no significant difference in survival between the groups (16 vs. 13 months, *P*=0.300).

**Conclusion:**

DCC is a safe and effective method for the management of OLA in lung transplantation.

**Table:** 

Abbreviations, acronyms & symbols				
6MWT	= Six-minute walk test		LTx	= Lung transplantation
BMI	= Body mass index		N/a	= Not available
COPD	= Chronic obstructive pulmonary disease		OLA	= Oversized lung allograft
CPB	= Cardiopulmonary bypass		PaO_2_	= Partial oxygen pressure
DCC	= Delayed chest closure		PAP	= Pulmonary artery pressure
ECMO	= Extracorporeal membrane oxygenation		PCC	= Primary chest closure
FEV1	= Forced expiratory volume in 1 second		PGD	= Primary graft dysfunction
FVC	= Forced vital capacity		pTLC	= Predicted total lung capacity
ICU	= Intensive care unit		PVR	= Pulmonary vascular resistance
ILD	= Interstitial lung disease		TAPSE	= Tricuspid annular plane systolic excursion
IPF	= Idiopathic pulmonary fibrosis			

## INTRODUCTION

Size matching is an important factor that directly affects the outcomes of lung transplantation. An oversized lung allograft (OLA) is defined as a donor/recipient predicted total lung capacity (pTLC) ratio > 1.0^[1,2]^. In practice, however, OLA identification can be used for any lung that does not fit into the thoracic cavity, with or without donor/recipient pTLC ratio > 1. The lungs are a highly susceptible organ and tend to be edematous, inflamed, and less compatible due to the effects of donor brain death, handling during procurement, cold storage, and ischemia-reperfusion injury^[3]^. Additionally, a dilated right heart and/or an elevated diaphragm and/or an excessive intrathoracic fat tissue may occupy a space in thoracic cavity^[4]^. Therefore, the lung allograft may become oversized due to one or more of these factors.

Primary closure of the chest after OLA use may cause undesirable complications such as atelectasis in the lungs, hemodynamic instability, primary graft dysfunction (PGD), venous occlusion, pneumonia, and anastomotic healing^[3-6]^.

Delayed chest closure (DCC) is one of the surgical maneuvering options that are administered to manage the size mismatch due to OLA. In plain definition, DCC is the closure of the open thorax without approaching the ribs and sternum. The primary goal here is to allow the lungs that do not fit into the thoracic cavity for various reasons to return to their original size and to prevent any compressive complications.

DCC is a well-defined method used in complicated cases of cardiac surgery since the 1970s^[7]^. However, it does not have a long history and extensive knowledge in lung transplantation^[6,8]^. Only a few serious case series have been published in the last decade^[4,8,9]^. The aim of our study was to evaluate DCC results after lung transplantation in our clinic.

## METHODS

### Patients

**Fig. 1:**
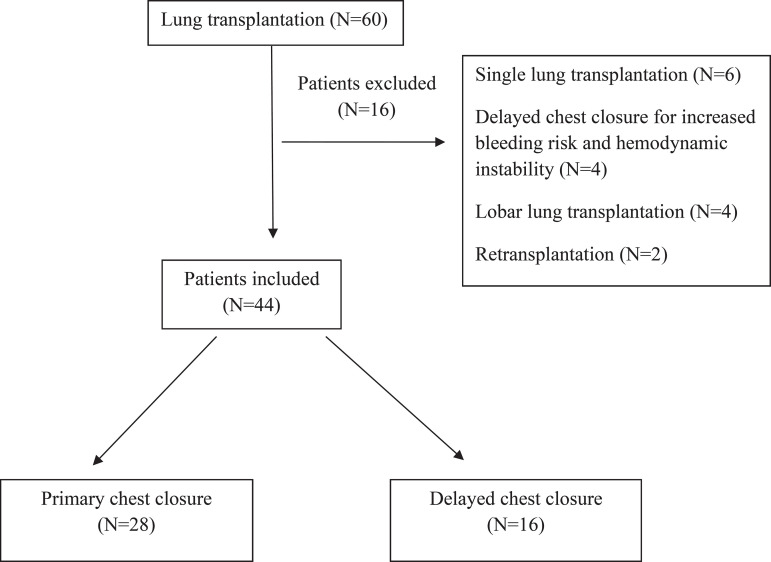
Patient selection.

Sixty patients who underwent lung transplantation between December 2016 and June 2019 were evaluated retrospectively. Patients who underwent bilateral lung transplantation and DCC for OLA were included in the study. Six patients who underwent single lung transplantation, four patients who underwent lobar transplantation, two patients who underwent retransplantation, and four patients who underwent DCC for hemodynamic instability were excluded from the study (Figure 1). Forty-four patients were divided into two groups as primary chest closure (PCC) (n=28) and DCC (n=16). Demographic characteristics, donor and operative characteristics, and results of the patients were compared.

### Surgical Technique and İntraoperative Data

Bilateral sequential lung transplantation was performed in all patients. The Clamshall incision was done through the 4^th^ or 5^th^ intercostal space. The lung was released. The donor lung was implanted after pneumonectomy and hilar dissection. Extracorporeal membrane oxygenation (ECMO) support was provided in cases of respiratory failure or hemodynamic deterioration. After bleeding control, two chest tubes were placed on both hemithoraces, and the thorax was closed. The DCC decision was made intraoperatively by the surgeon. In all patients, DCC was performed by simply closing the skin. The skin was approached with primary nonabsorbable sutures, and none of the patients required any material use or active sternal retraction. After DCC, the patients were transferred to intensive care unit (ICU), intubated, and followed up with mechanical ventilatory support. After two to four days of follow-up, the patients were revised under general anesthesia in the operating room. The skin was opened, and the graft was re-evaluated. In appropriate patients, the chest was closed. If necessary, the thorax was closed after diaphragmatic plication and/or lung resection. None of the patients had a second extension period of DCC. Graft ischemia time was defined as the time between insertion of aortic cross-clamp into the donor and initiation of allograft reperfusion. Total blood product amount was defined as total erythrocyte suspension and fresh frozen plasma and platelet solution units given to the patient in the first 24 hours after the beginning of the operation. Additional surgical procedure was defined as lung volume reduction resections (wedge resection and lobectomy) performed prior to final closure after surgery and widening of the thoracic cavity (diaphragm plication).

### Donor Characteristics

Donor information was obtained from the donor file. Donor age, gender, PaO_2_ level, and pTLC values were evaluated. pTLC was calculated using the formula presented by the European Respiratory Society^[10]^. According to this formula, $\mathrm{pTLC}\;\left(\mathrm{ml}\right)\;\mathrm{in}\;\mathrm{males}=7.99\;x\;\mathrm{length}\left(m\right)\;-\;7.08\;x\;1000\;\mathrm{and}\;\mathrm{pTLC}\left(\mathrm{ml}\right)\;\mathrm{in}\;\mathrm{females}=6.60\;x\;\mathrm{height}\;(m)\;-\;5.79\;x\;1000$ . These formulas were considered valid for ages between 18-70 and for lengths between and 1.55-1.95 m in men and 1.45-1.80 m in women.

### Postoperative Management and Follow-up

All patients received a standard triple immunosuppression regimen. Basiliximab induction was given at the days 0 and 4. Methylprednisolone, tacrolimus, and mycophenolate mofetil were applied as standard in all patients. Broad spectrum antibacterial, antifungal, and antiviral prophylaxis was given to all patients. Culture-sensitive antibiotics were preferred in patients with culture positivity. Antibacterial treatment was given for 10-14 days. All patients underwent routine clinical and bronchoscopic evaluations at two weeks and one, two, three, six, and 12 months.

Bleeding requiring re-exploration, tracheostomy, arrhythmia requiring treatment, bronchopleural fistula, renal insufficiency, dialysis, cerebrovascular event, and pleural empyema were defined as major complications. PGD and acute rejection were defined and graded according to the International Society for Heart and Lung Transplantation definition^[11,12]^. Grade 3 PGD and grade 2 acute rejection were defined as major complications. Wound infection was defined as superficial soft tissue infection without intrathoracic penetration. Patients who died within 90 days postoperatively were identified. Survival results were evaluated.

### Statistics

Statistical analysis was calculated using the IBM Corp. Released 2011, IBM SPSS Statistics for Windows, Version 20.0, Armonk, NY: IBM Corp program. Standard descriptive analyses were used for reporting results. Variables were expressed as mean ± standard deviation. Comparisons between groups were done using Mann-Whitney U test. P-value < 0.05 was defined as statistically significant. Kaplan-Meier test was used to calculate survival.

## RESULTS

### Patients’ Demographics

A total of 44 patients were included in the study. The mean age was 44.5 years and the male to female ratio was 25/19. The mean body mass index is 24.3 kg/m^2^. PCC was performed in 28 patients and DCC was performed in 16 patients. The mean DCC duration was three days (range, 2 to 4). There was no significant difference between the groups in terms of demographic characteristics (*P*>0.05). Although the mean oxygen use, six-minute walk test, and mean pulmonary artery pressure (or PAPm) were higher in DCC group, there was no significant difference between the groups (*P*>0.05). The most common indication in both groups was idiopathic pulmonary fibrosis/interstitial lung disease. The comorbidities were similar in both groups (Table 1).

**Table 1:** Patients’ demographics.

Parameter		Total (n=44)	PCC (n=28)	DCC (n=16)	*P*-value (<0.05)
	Age (years)	36.5±13.2	35.2±12.4	38.6±13.1	0.353
	Male	25	16	10	0.570
	Female	19	13	6	0.907
	BMI (kg/m^2^)	24.3±4.5	24.9±4.4	23.4±4.7	0.292
Respiratory state	Oxygen use at rest (lt)	4.2±2.0	3.8±1.6	4.9±2.4	0.077
	6MWT(m)	161±109	183±106	148±115	0.334
	FEV1 (%)	29.3±14.2	30.6±16.3	28±10.2	0.874
	FVC (%)	38.5±15.1	36.5±13.7	41.9±18.6	0.213
Cardiac state	Mean PAP (mm-Hg)	28.8±14.4	27.0±12.3	32.3±18.5	0.341
	TAPSE (mm)	2.0±0.6	2.2±0.5	2.0±0.8	0.235
	PVR (mm-Hg)	2.8±1.4	2.8±1.3	2.9±0.8	0.723
	Cardiac index (L/m/m^2^)	2.6±1.0	2.7±0.9	2.5±1.0	0.359
Indications	IPF	10	6	4	0.788
	ILD	13	8	5	0.853
	COPD	8	6	2	0.465
	Bronchiectasis	7	5	2	0.644
	Cystic fibrosis	5	3	2	0.859
	Sarcoidosis	1	0	1	0.186
Comorbidity	Diabetes mellitus	4	2	2	0.557
	Systemic arterial hypertension	6	4	2	0.870
	Previous thoracic surgery	9	6	3	0.834
	ECMO bridge to LTx	4	2	2	0.557
	Pleural empyema	1	0	1	0.186

6MWT=six-minute walk test; BMI=body mass index; COPD=chronic obstructive pulmonary disease; DCC=delayed chest closure; ECMO=extracorporeal membrane oxygenation; FEV1=forced expiratory volume in 1 second; FVC=forced vital capacity; ILD=interstitial lung disease; IPF=idiopathic pulmonary fibrosis; LTx=lung transplantation; PAP=pulmonary artery pressure; PCC=primary chest closure; PVR=pulmonary vascular resistance; TAPSE=tricuspid annular plane systolic excursion

### Donor Characteristics and İntraoperative Data

Donor age, gender, and PaO_2_ levels were similar in both groups. In the DCC group, donor/recipient pTLC ratio was significantly higher than in the PCC group (1.06 *vs*. 0.96, *P*=0.008). DCC was performed significantly more in donor/recipient pTLC ratio > 1.0 lung allografts than those in donor/recipient pTLC ratio ≤ 1.0 lung allografts (54% *vs*. 18%, *P*=0.013). Graft ischemia time, total operation time, total blood product use, intraoperative ECMO requirement, and need for additional surgical procedure were similar between the two groups (*P*>0.05).

### Postoperative Course, Complications, and Outcomes

In the DCC group, extubation time (4.3 *vs*. 3.1 days, *P*=0.002) and ICU length of stay (7.6 *vs*. 5.2 days, *P*=0.016) were significantly higher than in the PCC group. The incidence of major complications was similar in both groups and there was no significant difference between the total number of major complications (*P*>0.05). Postoperative wound infection was significantly higher in the DCC group compared to the PCC group (18.6% *vs*. 0%, *P*=0.019).

The 90-day mortality was 18% in the DCC group and 17% in the PCC group (*P*>0.05). Acute rejection episodes were observed in 31% of the DCC group and 39% of the PCC group at one-year follow-up (*P*>0.05). Median survival was 14 months in all patients. There was no significant difference in survival between the PCC and DCC groups (16 *vs*. 13 months, *P*=0.300) (Table 2).

**Table 2:** Donor characteristics, operative data, and outcomes.

Parameter		Total (n=44)	PCC (n=28)	DCC (n=16)	*P*-value
Donor age		35±12	33±12	38±14	0.175
Donor gender	Male donor	23	13	10	0.310
	Female donor	21	15	6	0.310
	Donor PaO_2_ (mm-Hg)	397±87	404±83	385±96	0.442
	Donor/recipient pTLC rate	0.99±0.1	0.96±0.1	1.06±0.8	0.008*
Allograft ischemia time	Ischemia of first lung (minute)	255±47	262±55	242±25	0.261
	Ischemia of second lung (minute)	420±56	422±63	416±44	0.582
	Total operation time (minute)	542±51	549±60	530±27	0.322
	Total blood product use	7.1±3.4	6.7±2.9	7.7±4.1	0.113
	Intraoperative ECMO use	17	9	8	0.822
Additional procedure	Wedge resection	6	4	2	0.870
	Lobectomy	4	4	0	0.117
	Diaphragm plication	5	2	3	0.249
	Excessive fat tissue excision	2	0	1	0.186
	Extubation time (day)	3.5±3.2	3.1±3.3	4.3±2.9	0.002*
	ICU time (day)	6.1±4.1	5.2±3.0	7.6±5.3	0.016*
Complications	PGD requiring ECMO	6	4	2	0.644
	Bleeding requiring re-exploration	2	2	0	0.279
	Tracheostomy	5	4	1	0.424
	Arrhythmia requiring treatment	6	4	2	0.870
	Bronchopleural fistula	1	1	0	0.450
	Renal insufficiency requiring dialysis	3	3	0	0.624
	Cerebrovascular event	1	1	0	0.450
	Pleural empyema	3	2	1	0.264
	Total major complication	27	21	6	0.312
	Wound infection	3	0	3	0.019*
	Acute rejection episode	16	11	5	0.598
	90-day mortality	8	5	3	0.942
	Median survival (mo)	14	16	13	0.300

DCC=delayed chest closure; ECMO=extracorporeal membrane oxygenation; ICU=intensive care unit; PaO2=partial oxygen pressure; PCC=primary chest closure; PGD=primary graft dysfunction; pTLC=predicted total lung capacity

* Significant P-value.

## DISCUSSION

Our results showed that DCC maneuver for bilateral lung transplantation is as safe and effective as PCC in terms of results. Although wound infection in DCC is more common than in PCC, and ICU length of stay and extubation time are longer in DCC than in PCC, early major complications, mortality, one-year acute rejection attacks, and survival were comparable with PCC.

Similar results have been reported in other important case series. Force et al.^[7]^ reported the first case series on DCC in 2006. In 28 lung transplantations, seven (25%) patients underwent DCC and were compared with 21 patients who underwent PCC. The mean DCC time was 5.3 days (range, 3 to 7). The DCC group showed significantly higher blood transfusion requirements, higher pulmonary artery pressure, greater cardiopulmonary bypass (CPB) usage, longer ischemia time, and a lower partial oxygen pressure/fraction of inspired oxygen, or PaO_2_/FiO_2_, ratio than the PCC group. PGD development and tracheostomy rate were higher and length of hospital stay was longer in the DCC group, but no difference was observed in one-month mortality. The authors argued that the method may be an important alternative strategy in patients with PGD findings. D’Cunha et al.^[13]^, between 2006 and 2008, performed DCC in five patients. One patient died due to bowel obstruction at five months. The average discharge time was 41 days (range, 26 to 62). The author reported that ECMO and its associated costs and serious complications can be avoided in unstable patients with DCC. In the largest case series of DCC published by Shigemura et al.^[4]^, 90 (10.3%) of 873 patients who underwent lung transplantation between 2004 and 2017 were performed DCC and their results were compared with the PCC group. The mean DCC duration is 4.5 days (range, 1 to 18). In DCC, operative time, CPB use, and duration and transfusion were higher. They reported that PGD requiring ECMO support, one-year acute rejection episode, and 30- and 90-day mortality were higher in the DCC group. In a study by Rafiriou et al.^[8]^, between 2009 and 2016, 51 (7%) of 770 patients who underwent lung transplantation were performed DCC and were compared with PCC. The average DCC duration was 4.6±2.3 days. The prolonged intubation was higher in the DCC group, but the results were similar in terms of other complications and long-term survival. Case series in the literature were given in Table 3.

**Table 3:** Case series in the literature.

Articles	Patients	Methods	Results	Conclusion
Force et al.^[7]^, 2006	- From January 2003 to March 2005	- Single-center retrospective cohort study	- Mean DCC time 5.3 days (3-7).	- DCC can be employed safely and outcomes are similar to PCC
	- Total 28 LTx	- Comparison of DCC *vs*. PCC	- In DCC:	- May also provide a treatment option for patients in whom PGD develops
	> DCC, N=8 (25%)	- DCC technique: Esmark bandaging in 7 and active sternal retraction in 1 patient	More tracheostomy	- May lead to a decreased mortality for this high-risk patient population
	> PCC, N=20		More hospitalization	
	- Indications N/a		More PGD	
			More CPB use	
			Longer CPB time	
			Similar infections	
			Operative mortality = 0%	
D'Cunha et al.^[13]^, 2010	- From October 2006 to February 2008	- Case series	- Mean DCC time 5.4 days (4-9)	- DCC is very favorable
	- 5 cases of DCC	- DCC technique: Esmarch dressing in all patients	- Mean hospital stay 41 days (26-62)	- Potentially avoids ECMO and its complications
	- Indications:		- No surgical infection	
	> Respiratory and hemodynamic instability, N=3		- No allograft failure	
	> Bleeding, N=2		- 19-month survival 80%	
Shigemura et al.^[4]^, 2014	- From January 2004 to December 2011	- Single-center retrospective cohort study	- Mean DCC time = 4.5 days (1-18)	- DCC can be safely performed with acceptable procedure-related risks
	- Total 873 LTx:	- Comparison of DCC *vs*. PCC	- In DCC:	- DCC should not be considered a sub-optimal option after LTx
	> DCC, N=90 (10.3%)	- Also comparison of DCC techniques	More operation time	- DCC strategies would contribute to decreasing the risk of PGD without increasing procedure-related risks
	> PCC, N=783	- DCC techniques:	More early postoperative bleeding	
	- Indications:	> Simple skin closure (DCC-1), N=52	More PGD	
	> Acute lung edema, N=40	> Esmark bandage (DCC-2), N=30	More acute rejection	
	> OLA, N=38	> Active sternal retraction with rib spreader (DCC-3), N=8	More 30- and 90-day mortality	
	> Coagulopathy/bleeding, N=29		No more infection	
	> Hemodynamic instability, N=18		- In technical comparison:	
			> DCC-1 similar to PCC	
			> Decreases PGD (9.6% *vs*. 26%)	
			> Improve survival and functional status	
			> DCC-2 and DCC-3 increase mortality	
Aguilar et al.^[9]^, 2017	- From January 1 2010 to July 31 2014	- Single-center retrospective cohort study	- Median DCC time = 2 days.	- DCC is an independent risk factor for surgical site infection after LTx
	- 232 LTx	- Comparison of DCC *vs*. PCC.	- In DCC:	- DCC is necessary in selected patients
	> DCC, N=67 (29%)	- Technique:	More infection (19% *vs*. 5%)	
	> PCC, N=165	> Simple skin closure, N=59	More grades 2 and 3 PGD	
	- Indications:	> Rubber fish device to cover the wound, N=8	More intraoperative CPB	
	> Bleeding		More ischemic time	
	> OLA		More ICU time	
	> Severe pulmonary edema		Similar mortality	
	> Hemodynamic instability			
Rafiroiu et al.^[8]^, 2018 (Abstract)	- From January 2009 to January 2016.	- Single-center retrospective cohort study	- Mean DCC time = 4.6±2.3 days	- Patients requiring DCC represent a high-risk group of patients undergoing LTx
	- 770 LTx	- Comparison of DCC *vs*. PCC	- In DCC:	- DCC is not associated with increased risk of infection, morbidity, and mortality
	> DCC, N=51 (7%)	- Technique:	No more infection	
	> PCC, N=719	> A composite material use	Prolonged intubation	
	- 47 pairs of DCC and PCC patients were included according to a greedy matching algorithm.		More stroke	
	- Indications:		More permanent dialysis	
	> Severe coagulopathy		Similar survival	
	> Intolerance to PCC due to hypoxia or cardiac tamponade			
Yeginsu et al.	- From December 2016 to January 2019	- Single-center retrospective cohort study	-Mean DCC time = 3 days (2-4).	- DCC is a safe and effective option in the management of size mismatch due to OLA
	- 60 LTx	- Comparison of DCC *vs*. PCC	- In DCC:	- DCC may be associated with increased risk of infection
	> 20 DCC (33%)	- Technique:	Prolonged extubation time	- Further studies are needed to evaluate the value of other options in the management of size mismatch as well
	> 40 PCC	> Simple skin closure	Prolonged ICU time	
	- Excluded, N=16		More wound infection	
	- 16 DCC and 28 PCC were included		No more major complications	
	Indications:		No more acute rejection	
	> Only OLA		Similar median survival	

CPB=cardiopulmonary bypass; DCC=delayed chest closure; ECMO=extracorporeal membrane oxygenation; ICU=intensive care unit; LTx=lung transplantation; N/a=not available; OLA=oversized lung allograft; PCC=primary chest closure; PGD=primary graft dysfunction

In addition to OLA, DCC may also be required in cases of acute pulmonary edema, hemodynamic instability, and high risk of bleeding^[4,9]^. The indication for DCC depends on the choice of the surgeon intraoperatively. In OLA, there is a size mismatch, which needs to be handled intraoperatively. In this PCC case, the surgeon has two options: allograft volume reduction (lung resections) and/or expansion of the thoracic volume (excision of excessive intrathoracic fat tissue and diaphragmatic plication). However, surgical resection of lung volume should always be the last resort. Instead, DCC maneuver may be certainly a more reasonable solution to allow the lung to shrink spontaneously. Thus, the allograft can reach its normal size and the problem can be solved with a smaller process. In our clinic, we routinely perform DCC first in the management of OLA. After two to four days of waiting period, if the lungs fit into the thoracic cavity, we do PCC. If the lungs still do not fit, we first perform maneuvers (diaphragm plication and/or excision of excessive intrathoracic fat tissue) to enlarge the thoracic cavity. If the lungs still do not fit, resection is the last resort. We did not need lobectomy in any of the patients who underwent DCC with this application.

Technically, it is possible to perform three methods for DCC after lung transplantation. Type 1, simple skin approximation using continuous suture^[4]^; Type 2, the chest is left open and a layer of latex-free Esmark bandaging (Fulflex Elastomerics Worldwide, Lincoln, Rhode Island, United States of America) is attached to the skin using sutures^[4,7,13]^; and Type 3, the sternum is retracted by a rib spreader and then the second method is performed^[4,7]^. We always used simple skin approximation in all patients. We never needed the other two types of method. Shigemura et al.^[4]^ reported that Types 2 and 3 DCC techniques were associated with a higher risk of death, whereas in the Type 1 DCC technique, the risk of PGD requiring ECMO, renal insufficiency, and death was reduced compared to PCC. In multivariate analysis, prolonged CPB duration, postoperative ECMO requirement, and Type 3 DCC technique increased the risk of death in DCC. The findings of Shigemura partially explain why our results are slightly better than of some other reports.

Whether DCC increases the risk of infection is controversial. Aguilar et al.^[9]^ performed DCC in 67 (29%) of 232 patients who underwent lung transplantation between 2010 and 2014. In 22 (9%) of the transplanted patients, infection developed at the surgical site. Eighteen of them were wound infection, eight were pleural infection, and four were concomitant wound and pleural infection. Patients with DCC had significantly more infections than those with PCC (19% *vs*. 5%, *P*=0.001). In multivariate analyses, DCC was found to be an independent risk factor for surgical site infections. However, Force et al.^[7]^, Shigemura et al.^[4]^, and Rafiriou et al.^[8]^ reported in their published case series that DCC did not increase the risk of infection. In our case series, surgical site infection (three wound infections and three pleural empyemas) was detected in six (10%) patients. Supporting the results of Aguilar, only wound infection was significantly higher in patients undergoing DCC compared to PCC patients (18% *vs*. 0%, *P*=0.019).

The use of OLA has been reported to reduce the risk of PGD and increase long-term survival after lung transplantation^[1,2]^. However, Shigemura et al.^[4]^ reported that after a lung transplant using an OLA, the primary PCC disrupted the allograft hemodynamics and physiology, exacerbating existing lung injury and increasing the risk of developing PGD. They reported that the development of PGD requiring ECMO support was significantly higher in patients undergoing DCC than in those undergoing PCC (31% *vs*. 2.8%, *P*<0.05). However, the development of PGD varied with the technique of DCC. PGD development rate after DCC with simple skin closure technique (Type 1) was 9.6%, and it was 60% and 62% for the second and third techniques (Types 2 and 3), respectively. The authors concluded that PGD can be reduced by appropriate technique and careful post-DCC management. However, they did not explain why DCC with the Type 1 technique leads to less PGD. Force et al.^[7]^ detected moderate or severe PGD findings in five of the six patients who underwent DCC and the frequency was significantly higher than that of PCC (*P*=0.0002). Aguilar et al.^[9]^ found that postoperative grades 2 and 3 PGD development was higher in patients with DCC compared to those with PCC. In our case series, total PGD was 10% and there was no significant difference between the groups. The fact that our DCC case series covers only OLA and that we used only simple skin closure technique (Type 1) in all patients may have affected our results.

The timing and criteria of final closure of the chest of patients undergoing DCC are not fully defined. The average DCC duration is 4.5 to 6 days (range, 1 to 18) in publications^[4,7-9,13]^. In DCC lasting more than three days, exploration and washout are performed in every three days in the operating room and this is continued until final closure. Aguilar et al.^[9]^ reported median DCC duration as two days and median washout number as one. In our practice, the average DCC time was three days (range, 2 to 4) and no patient needed recurrent explorations. At the end of the DCC period, the chest was reopened, and exploration and washout were performed. The criteria for final closure after DCC are not well defined. D’Cunha et al.^[13]^ think that it is sufficient to have a central venous pressure > 10 mmHg and an acceptable low hemodynamic support for final closure. Shigemura et al.^[4]^ recommend that the patients have good renal function and diuresis, the allograft is dry, the coagulation parameters are normal, and the DCC duration is > 72 hours. Also, they recommend being patient and do not rush for final closure. Although there are no definite indications for final closure after DCC in our clinic, it is generally sufficient for us to have dry graft on chest radiography and to be hemodynamically stable. However, we agree with Shigemura's recommendation that the final closing should be done at least 72 hours later.

## Limitations

This is a single-center retrospective cohort study and the data were taken from the patients’ files. Therefore, the data of some patients may be overlooked or not objective enough. Since the sample size was small, a general statistical comparison was made, and detailed statistical models could not be studied. However, only patients undergoing DCC for OLA were included in the study and the cohort was attempted to be homogenized.

## CONCLUSİON

We believe that DCC contributes to the solution of the problem of mismatch caused by OLA in lung transplants without increasing the major complications and mortality. However, further studies are needed to compare DCC with other applications in the management of mismatching caused by OLA.

**Table:** 

Authors' roles & responsibilities	
AY	Substantial contributions to the conception or design of the work; or the acquisition, analysis, or interpretation of data for the work; drafting the work or revising it critically for important intellectual content; agreement to be accountable for all aspects of the work in ensuring that questions related to the accuracy or integrity of any part of the work are appropriately investigated and resolved; final approval of the version to be published
AET	Drafting the work or revising it critically for important intellectual content; final approval of the version to be published
MV	Drafting the work or revising it critically for important intellectual content; final approval of the version to be published
BA	Substantial contributions to the conception or design of the work; or the acquisition, analysis, or interpretation of data for the work; final approval of the version to be published
NH	Final approval of the version to be published
AE	Final approval of the version to be published
SC	Substantial contributions to the conception or design of the work; or the acquisition, analysis, or interpretation of data for the work; final approval of the version to be published
EC	Drafting the work or revising it critically for important intellectual content; final approval of the version to be published
